# Psychosocial burden, vaccination status and preventive information options for seafarers during the COVID-19 pandemic

**DOI:** 10.1038/s41598-025-20616-3

**Published:** 2025-09-23

**Authors:** Nora M. Puls, Benedict Oldenburg, Chiara Reck, Dorothee Dengler, Lukas Belz, Lorenz Scheit, Volker Harth, Marcus Oldenburg

**Affiliations:** 1 University Medical Center Hamburg-Eppendorf, Seewartenstr. 10, 20459 Hamburg, Germany; 2 Clinic I-Internal Medicine, Bundeswehr Hospital Hamburg, Lesserstr. 180, 22049 Hamburg, Germany

**Keywords:** Seafarers, COVID-19, Mental health, Knowledge, Vaccination, Occupational health, Public health

## Abstract

The vaccination rate of seafarers during the COVID-19 pandemic was initially low. Along with the well-known crew change crises, it likely placed a high psychosocial burden on shipping crews. The aim of this work is to assess the vaccination rate, determine triggers for psychosocial symptoms and emphasize preventive-psychological information channels and media for seafarers. In March 2022, 583 seafarers and 24 office workers from the same shipping company were surveyed by using a questionnaire. It included the standardized Fear of Coronavirus Questionnaire and further questions on COVID-19. A total of 479 (81.3%) participants had been vaccinated. However, office workers were 31 times more likely to have received a basic immunization with one booster vaccination than seafarers (CI 12.0-81.9). Seafarers had significantly more often fear of Corona-related health consequences than office workers (73.9% vs. 41.7%, OR 4.0, CI 1.7–9.1) – also, ratings 2.5 times more than officers (81.2% vs. 63.3%; *p* < 0.001). In addition, seafarers – and among them the ratings in particular – rated the pandemic burden regarding social and financial impacts significantly higher. Although 75% of seafarers felt (very) well informed about COVID-19 issues and vaccination, they were 20.9 times more interested in further information than office workers (CI 2.8-155.5). For all seafarers, the shipping company, ship personnel and international organizations were the most regularly used sources of information. This study indicates that the COVID-19 vaccination rate was lower among seafarers than the shore-based population. This can lead to increased pandemic-related psychosocial stress. Improving the dissemination of information could contribute to better coping strategies in seafaring. Overall, these findings highlight the importance of supporting seafarers in future crises and the need to improve international strategies for the treatment of seafarers as key workers.

**Supplementary Information:**

The online version contains supplementary material available at 10.1038/s41598-025-20616-3.

## Background

 The COVID-19 pandemic was by now the greatest pandemic in this century. In January 2020, the World Health Organization (WHO) declared this outbreak of coronavirus disease a “Public Health Emergency of International Concern (PHEIC)”. This classification was only lifted again in May 2023^1^. By April 2024, more than 700,000,000 people had been tested positive for the COVID-19 virus – far more if unregistered cases are taken into account – and around 7 million people had died from the virus by then^2^. The pandemic caused numerous restrictions in social life, health care as well as traveling and was the cause for a major social and economic crisis worldwide.

As the shipping industry covers more than 90% of the world trade, including medical goods, raw materials and foods^3,4^, many key industry associations underlined the importance of improving crew changes, designating seafarers as key workers and prioritizing their vaccination^5,6^. Many countries and flag states supported this call, but ultimately local regulations prevailed. Although the International Chamber of Shipping published a “Roadmap for Vaccination of Seafarers”^7^, the progress on vaccination was very slow^8,9^. According to a survey of the Global Maritime Forum only 15% of the seafarers were vaccinated by August 2021, 89% by July 2022 and 97% by July 2023^9^. However, more data on the vaccination status of seafarers is needed, as no central tracking has been established and consequently approved numbers are still lacking^9^.

For the shipping industry the pandemic-related restrictions resulted in a “crew change crisis” – around 400,000 seafarers were stranded at sea in 2020^10,11^ – and a deterioration in the already challenging working conditions^12,13^. This situation not only led to a higher risk for accidents^14,15^, but also had various effects on the wellbeing of ship crews: It has been widely described that the pandemic had a negative impact on the mental health of seafarers^8,15–17^. In particular, Pausztat et al. show that seafarers developed more anxiety and depressive symptoms during the pandemic than before and that these symptoms were also related to length of stay^18^. To date, the triggers for the increase in psychological symptoms have not been fully researched. Seafarers faced the risk of financial insecurities due to the inability to attend work and the fear of future unemployment due to blacklisting if they raised their voices against the extensions of contracts beyond the permitted maximum^8,16,17,19–22^. In addition, the COVID-19 virus affected seafarers’ social wellbeing^8,19,21^; there were restrictions on personal contact worldwide, but seafarers had to live and work closely together, leading to an increased risk of virus transmission^23,24^. Accordingly, Coutroubis et al. observed that about 90% of ship personnel were afraid of an infection, somebody else bringing it onboard and traveling home after their stay^20^.

Knowledge is an important factor in coping with anxiety and effective specialist information can effectively reduce the triggers of psychological symptoms in terms of a preventive psychological approach^25^. It is also important to note that there is little data on knowledge of COVID-19 among seafarers: Battineni et al. sent a questionnaire to 5000 seafarers in 2020 of which 1458 responded. It contained six knowledge questions (two on clinical presentation, two on transmission routes and two on prevention). The authors analysed an overall percentage of 97% correct answers, with no difference between educational groups^26^. Although these results demonstrate a high level of objective knowledge among seafarers, there is still a lack of information on which channels and media seafarers typically use to obtain expert information e.g. about the COVID-19 pandemic and whether their information needs have been adequately met.

The pandemic has had a tremendous impact on the well-being of seafarers, and the risk of further pandemics in the coming decades is ubiquitous – if not increasing –^27^. To draw lessons from the past pandemic, the present study aims to identify vaccination rates among shipping crews and reasons for not being vaccinated in pandemic times. Furthermore, it intends to determine the triggers for psychosocial symptoms among seafarers as well as evaluate preventive psychological information ways for crews on pandemic-related topics.

## Methods

The cross-sectional study was conducted by using an Englisch-language questionnaire as part of a broader survey on health management on board. The questionnaire was handed out in February 2022 to all seafarers (*n* = 616) and all office workers (*n* = 62), as an internal reference group, of the same Hamburg shipping company. There were no exclusion criteria. It was sent by email to the captains of the ships – or to a person in the company – printed out, forwarded to the seafarers on board, collected within two weeks without direct contact, scanned collectively per ship and finally emailed back to the researchers. To minimize human error in data entry, the data was processed according to the dual control principle and plausibility checks were carried out.

At the beginning of the survey, participants were informed in writing that their data would be treated confidentially and anonymously and that their participation was voluntary. Prior to the study, the participants were verbally familiarized with these aspects and received detailed information about the survey procedure and the security mechanisms to ensure the anonymity of the data (especially vis-a-vis employers). It was also explicitly pointed out that participants had the option of leaving certain questions unanswered or withdrawing their consent at any time. In this way, verbal informed consent was ensured, which was also approved by the responsible ethics committee (Hamburg Medical Chamber (Ethik Kommission der Hamburger Ärztekammer)) (PV7174). All methods were performed in accordance with the relevant guidelines and regulations. The report on this questionnaire was based on the Consensus-Based Checklist for Reporting Survey Studies (CROSS)^39^. This checklist can be found as Supplementary Table S1 online.

The survey consisted of 37 questions, 9 of which related to the COVID-19 pandemic. The questions that were evaluated for this study can be found as Supplementary Material S2 online. As there were no mandatory questions, there is missing data for some questions. To avoid having to exclude too much data, these were omitted from the specific questions. In particular, 31 participants did not indicate their rank, which is why they were not included in the group comparisons between officers and ratings, but were included in the group of seafarers as a whole.

### Vaccination status

Respondents were asked to provide information about their vaccination status as well as the frequency and type of vaccinations they had received. In addition, the shipping crews were asked to state their reasons for not being vaccinated against Coronavirus in free text.

### Fear of the coronavirus questionnaire (FCQ)

The seafarers’ subjective perception of their fear of COVID-19 was assessed using the standardized Fear of the Coronavirus Questionnaire (FCQ). This was developed by Mertens et al. in 2020^28,29^. It is an 8-item questionnaire based on different dimensions of fear (e.g., subjective worry, safety behaviours, preferential attention), with seafarers rating their level of agreement with each statement on a 5-point Likert scale (ranging from 1 = “Strongly disagree” to 5 = “Strongly agree”). By adding up the items, a total score can be calculated that ranges from 8 to 40 points, with higher scores indicating a greater fear of the coronavirus. In previous studies, a reliable scale was measured (Cronbach’s alpha = 0.75–0.80)^28–30^.

In addition to the assessment of fear of COVID-19, questions were asked about the subjectively perceived burden of financial and social consequences - also as potential triggers for psychosocial symptoms. A 6-point Likert scale (ranging from 0 = “Not at all” to 5 = “Extremely” was used here.

### Information channels and media

Since the relevance of triggers essentially depends on individual knowledge about the specifics of a threat situation and its risks^25,31^, it is crucial to understand and improve the dissemination of (scientifically based) information. Therefore, seafarers were asked via single-choice questions (“very poorly informed”, “poorly informed”, “fairly informed”, “well informed”, “very well informed”) whether they felt sufficiently informed about COVID-19 issues and vaccines, as well as about information channels and media related to COVID-19. A free-text question allowed participants to complete the given list about information channels and media.

### Statistics

Statistical analysis was performed with IBM SPSS Statistics 29, using a significance level α of 5% (*p* ≤ 0.05). The Pearson Chi-Quadrat test was used to calculate group differences for dichotomous variables. The T-test for unequal variances was used to determine differences between groups and interval scaled variables. This is in line with the recommendations of Rasch et al. and Ruxton that the assessment of assumptions beforehand can cause unknown type-I- and type-II-risks and that the Welch-test is the preferrable statistical instrument instead of the t-test and the Mann-Whitney U test^32,33^. In the case of significant group differences, the odds ratios were calculated using binominal logistic regression analysis (CI 95%). An adjustment was also made for important demographic/occupational variables (age, gender, ethnicity, education). For the variables that were not dichotomous, a median split was performed beforehand, and the median value was included in the calculations. In addition, the Cohen’s d value was calculated, due to correspondingly high odds ratios, for the differences in vaccination doses, FCQ scores and interest in further information about COVID-19.

## Results

### Demographic and occupational data

Of the total of 678 surveys distributed, 607 (89.5%) returned – 583 (94.6%) of the seafarers and 24 (38.7%) of the office workers. Due to the anonymous nature of the survey, it was retrospectively not possible to evaluate reasons for non-participation. Of a total of 24 office workers 17 (70.8%) were male and 7 (29.2%) female. 8 (33.3%) of those were aged < 40 years and 16 (66.7%) ≥ 40 years. In comparison, of 583 seafarers 541 (95.2%) were male, 22 (3.9%) female and 5 (0.9%) did not specify. 381 (67.1%) were < 40 years, 187 (32.9%) ≥ 40 years. 242 (43.7%) seafarers were European, 249 (44.9%) Asian and 63 (11.4%) American.

Of 583 seafarers 215 (38.9%) were officers and 337 (61.1%) ratings. 197 officers (93.8%) were male, 11 (5.2%) female and 2 (0.9%) did not specify. In comparison 318 (96.7%) ratings were male, 10 (3.0%) female and 1 (0.3%) did not specify. European 138 (66.0%), Asian 51 (24.4%) and 20 (9.6%) of the officers and 89 (27.7%), 193 (60.1%) and 39 (12.1%) of the ratings. Of the officers 31 (14.9%) had less than a high school degree, compared to the ratings with 299 (77.6%). Detailed demographic data are displayed in Table 1.

**Table 1:** Demographic data on the occupational subgroups.

Characteristic	Office Workers* (*n* = 24)	Seafarers* (*n* = 583)	Officers* (*n* = 215)	Ratings* (*n* = 337)
*Sex*				
Male	17 (70.8%)	541 (95.2%)	197 (93.8%)	318 (96.7%)
Female	7 (29.2%)	22 (3.9%)	11 (5.2%)	10 (3.0%)
Not Specified	0	5 (0.9%)	2 (0.9%)	1 (0.3%)
*Age*				
19 years or younger	0	1 (0.2%)	1 (0.5%)	0 (0%)
20–29 years	0	164 (28.9%)	34 (16.0%)	126 (37.7%)
30–39 years	8 (33.3%)	216 (38.0%)	108 (50.9%)	99 (29.6%)
40–49 years	5 (20.8%)	97 (17.1%)	40 (18.9%)	54 (16.2%)
50–59 years	8 (33.3%)	75 (13.2%)	21 (9.9%)	49 (14.7%)
60 years or more	3 (12.5%)	15 (2.6%)	8 (3.8%)	6 (1.8%)
*Origin*				
European	24 (100%)	242 (43.7%)	138 (66.0%)	89 (27.7%)
Asian	0	249 (44.9%)	51 (24.4%)	193 (60.1%)
American	0	63 (11.4%)	20 (9.6%)	39 (12.1%)
*Rank*				
Officer	---	215 (38.9%)	---	---
Rating	---	337 (61.1%)	---	---
*Years as a seafarer*	---	12.6 ± 9.5	14.5 ± 9.2	11.3 ± 9.6
*Education*				
Less than high school degree	7 (29.2%)	269 (51.2%)	31 (14.9%)	229 (77.6%)
High school degree or more	17 (70.8%)	256 (48.8%)	177 (85.1%)	66 (22.4%)
*Family status*				
Single	4 (17.4%)	182 (32.9%)	40 (18.6%)	134 (39.8%)
Partnership	19 (82.6%)	371 (67.1%)	164 (76.3%)	190 (58.6%)
Children	17 (70.8%)	342 (60.6%)	145 (68.7%)	180 (54.9%)

* The number of analysed answers differs per question category deviating from the basic population.

### Vaccination status

As of February 2022, a total of 479 (81.3%) participants were vaccinated. Although 23 (95.8%) office workers received a vaccination compared to 456 (80.7%) of the seafarers, there is no significant difference (*p* = 0.063). Additionally, there were no significant differences regarding ranks (ratings vs. officers) or cultural groups (Europeans vs. non-Europeans) (*p* = 0.230, *p* = 0.113). In contrast, the number of doses received at this point of time differs significantly (*p* < 0.001). While 7 (30.4%) office workers had been vaccinated twice and 16 (69.6%) three times, 313 (66.6%) seafarers had been vaccinated twice and 31 (6.8%) three times as demonstrated in Table 2. Office workers received 31 times more often a basic immunization with one booster vaccination, equal to three vaccine doses, than seafarers (CI 12.0-81.9, Cohen’s d 1.65, CI 1.22–2.08)^34^. There are no differences between officers and ratings in vaccination rate or doses.

**Table 2:** Differences of COVID-19 vaccination and vaccination doses between seafarers and office workers.

COVID-19 vaccination	Total* (*n* = 607)	Seafarers* (*n* = 583)	Office* workers (*n* = 24)	*p*	Crude OR (95% CI)
Received	479 (81.3%)	456 (80.7%)	23 (95.8%)	0.063	
*Doses*				**< 0.001**	
One dose	112 (23.4%)	112 (24.6%)	0		
Two doses	320 (66.8%)	313 (66.6%)	7 (30.4%)		
Three doses	47 (9.8%)	31 (6.8%)	16 (69.6%)		31.3** (12.0-81.9)

* The number of analysed answers differs per question category deviating from the basic population. ** OR remained significant after adjustment for age, sex, ethnicity and education Significant findings are in bold.

As reasons for no vaccination 41 seafarers (7%) stated that there was no availability for vaccination. Furthermore, 12 seafarers feared side effects and 8 seafarers were sceptical about the efficacy of vaccination. In addition, 8 seafarers declared not to have had time for vaccination and 2 stated that there was no compulsory vaccination in their home country.

### Health-related, social and financial concerns

Seafarers had a significantly greater fear of health consequences compared the office workers (mean value 28.7 ± 6.6 vs. 23.4 ± 5.7; *p* < 0.001) referring to the Fear of the Coronavirus Questionnaire. This corresponds to the finding that seafarers feared these health consequences nearly four times more often than office workers (CI 1.4–10.4; Cohen’s d 0.80, CI-0.39-1.21). Generally, seafarers significantly stocked more up on supplies, found the virus to be much more dangerous for their personal health, felt more often that health authorities are not doing enough to deal with the virus and took more precautions to not become infected than office workers (Table 3). Furthermore, a significant difference was found regarding ranks as ratings experienced more often fear of health consequences than officers (mean value 30.4 ± 6.1 vs. 26.2 ± 6.5; *p* < 0.001). In regard of the Odds ratio, ratings had 3.7 times more often fear of health consequences than officers (CI 2.6–5.4, Cohen’s d 0.67, CI 0.49–0.85) and stated a significantly higher rate in all questions of the FCQ-questionnaire than officers (Table 3).

Another difference between seafarers and office workers is the feeling of being burdened by social and financial consequences. The seafarers rated the pandemic burden regarding social impacts with a mean value of 3.5 (± 1.4) and regarding financial impact with a mean value 3.6 (± 1.3). This differs significantly to the office workers in light of social consequences (2.5 ± 1.6) and financial consequences (1.7 ± 1.5). In respect of occupational groups, ratings experienced the social and financial impact 2.2 times and 2.9 times higher than officers (CI 1.5–3.4, CI 1.9–4.4). These results are shown in Table 4.

### Information channels and media

Regarding the knowledge on the pandemic, 17 (3.0%) of the seafarers feel (very) poorly informed, 81 (14.3%) fairly informed and 469 (82.7%) well or very well informed about COVID-19 issues. None of the office workers feels very poorly or poorly informed. The mean value of the seafarers is 3.1 (± 0.8) and 2.9 (± 0.6) of the office workers. Referring to the feeling of being informed about COVID-19 vaccinations, 40 (7.0%) of the seafarers feel (very) poorly informed, 98 (17.3%) fairly informed and 430 (75.7%) well or very well informed. None of the office workers feels very poorly or poorly informed. The mean value of the seafarers is 3.0 (± 0.9) and 3.0 (± 0.6) of the office workers. Ratings felt about twice better informed about COVID-19 issues or COVID-19 vaccination than officers (CI 1.3–2.8, CI 1.3–2.9) (Table 4).

Concerning the information media used to disseminate facts on COVID-19 by work environment seafarers used significantly more often messenger platforms than office workers. Although only 1.6% of the shipping crews, in contrast to 13% of the office workers, didn’t receive information, they were 20.9 times more often interested in further information about the Coronavirus (CI 2.8-155.5, Cohen’s d 1.27, CI 0.86–1.69). Related to the occupational groups, ratings used 1.9 times more often messenger platforms as communication channels and were 2.9 times more interested in receiving further information about the Coronavirus than officers (CI 1.4–2.8, CI 2.0-4.2) (Table 5).

**Table 3:** FCQ-questionnaire and differences between the subgroups.

	Office workers* (*n* = 24)	Seafarers* (*n* = 583)	*p*	Crude OR (95% CI)	Officers* (*n* = 215)	Ratings* (*n* = 337)	*p*	Crude OR (95% CI)
**Fear of health consequences** **(FCQ-questionnaire);** total score (mean value ± SD)	23.4 ± 5.7	28.7 ± 6.6	**< 0.001**	3.8 (1.4–10.4)	26.2 ± 6.5	30.4 ± 6.1	**< 0.001**	3.7** (2.6–5.4)
I am very worried about the corona virus outbreak	2.8 ± 1.2	3.5 ± 1.2	**0.014**	5.8 (0.8–43.5)	3.1 ± 1.2	3.8 ± 1.1	**< 0.001**	3.3** (2.0-5.5)
I am taking precautions to prevent infection (e.g., washing hands, avoiding contact with people, avoiding door handles)	4.0 ± 0.9	4.2 ± 0.9	0.145		4.1 ± 0.9	4.3 ± 0.9	**< 0.001**	2.3** (1.6–3.3)
I am constantly following all news updates regarding the virus	3.4 ± 1.1	3.6 ± 1.1	0.504		3.2 ± 1.1	3.8 ± 1.0	**< 0.001**	2.9 (1.8–4.9)
I have stocked up on supplies to prepare for problems related to the coronavirus outbreak	2.2 ± 1.1	3.3 ± 1.1	**< 0.001**	5.5** (1.6–18.8)	2.9 ± 1.1	3.6 ± 1.1	**< 0.001**	2.5 (1.8–3.6)
For my personal health I find the virus to be much more dangerous than the seasonal	2.5 ± 1.3	3.4 ± 1.1	**< 0.001**	4.6 (0.6–34.1)	3.1 ± 1.2	3.7 ± 1.1	**< 0.001**	3.7 (2.1–6.5)
I feel that the health authorities are not doing enough to deal with the virus	2.6 ± 1.0	3.2 ± 1.1	**0.004**	3.4** (1.2–10.2)	3.0 ± 1.1	3.3 ± 1.1	**0.004**	2.0** (1.4–2.9)
I am worried that friends or family will be infected	3.4 ± 1.1	3.9 ± 1.1	**0.012**	3.6** (1.1–12.1)	3.7 ± 1.1	4.1 ± 1.0	**< 0.001**	2.2** (1.5–3.2)
I take more precautions compared to most people to not become infected	2.6 ± 1.0	3.7 ± 1.1	**< 0.001**		3.3 ± 1.1	3.9 ± 1.1	**< 0.001**	3.9** (2.4–6.2)

* The number of analysed answers differs per question category deviating from the basic population. ** OR remained significant after adjustment for age, sex, ethnicity and education Significant finding are in bold.

**Table 4:** Pandemic related burden by social and financial impact, knowledge of COVID-19 issues and vaccination.

	Office workers* (*n* = 24)	Seafarers* (*n* = 583)	*p*	Crude OR (95% CI)	Officers* (*n* = 215)	Ratings* (*n* = 337)	*p*	Crude OR (95% CI)
**Pandemic related burden as a consequence of…** (mean value ± SD on a scale from 0 to 5 (extremely burdened))								
…social impact	2.5 ± 1.6	3.5 ± 1.4	**0.005**	2.8 (0.8–9.5)	3.3 ± 1.4	3.8 ± 1.3	**< 0.001**	2.2** (1.5–3.4)
…financial impact	1.7 ± 1.5	3.6 ± 1.3	**< 0.001**	10.1 (1.3–75.1)	3.4 ± 1.3	3.8 ± 1.3	**< 0.001**	2.9** (1.9–4.4)
**Feeling sufficiently informed about…** (mean value ± SD on a scale from 1 to 5 (= very well informed))								
…COVID-19 issues	2.9 ± 0.63	3.1 ± 0.8	0.126		2.9 ± 0.8	3.2 ± 0.7	**< 0.001**	1.9 (1.3–2.8)
→ (very) poorly informed	0	17 (3.0%)			7 (3.4%)	8 (2.4%)		
→ fairly informed	6 (25%)	81 (14.3%)			45 (21.4%)	31 (9.4%)		
→ (very) well informed	18 (75%)	469 (82.7%)			158 (75.3%)	291 (88.1%)		
…COVID-19 vaccination	3.0 ± 0.6	3.0 ± 0.9	0.993		2.8 ± 0.9	3.1 ± 0.8	**< 0.001**	2.0 (1.3–2.9)
→ (very) poorly informed	0	40 (7.0%)			20 (9.4%)	14 (4.9%)		
→ fairly informed	5 (21.7%)	98 (17.3%)			46 (21.8%)	48 (14.6%)		
→ (very) well informed	18 (78.3%)	430 (75.7%)			145 (68.8%)	265 (80.5%)		

* The number of analysed answers differs per question category deviating from the basic population. ** OR remained significant after adjustment for age, sex, ethnicity and education. Significant findings are in bold

**Table 5:** Information media as well as the interest of receiving further COVID-19 information.

	Office workers* (*n* = 24)	Seafarers* (*n* = 583)	*p*	Crude OR (95% CI)	Officers* (*n* = 215)	Ratings* (*n* = 337)	*p*	Crude OR (95% CI)
Information media used to disseminate facts on COVID-19 by work environment								
Person-to-person communications	13 (56.5%)	374 (66.0%)	0.350		134 (62.3%)	221(68%)	0.174	
E-mails	13 (56.5%)	281 (49.6%)	0.513		132 (61.4%)	135 (41.5%)	**< 0.001**	0.4** (0.3–0.6)
Flyers/leaflets	3 (13%)	69 (12.2%)	0.900		29 (13.5%)	33 (10.2%)	0.234	
Web-based trainings	1 (4.3%)	111 (19.6%)	0.068		34 (15.8%)	71 (21.8%)	0.083	
Messenger platforms	4 (17.4%)	277 (48.9%)	**0.003**	4.5 (1.5–13.5)	85 (39.5%)	182 (56.0%)	**< 0.001**	1.9** (1.4–2.8)
“I didn’t receive information”	3 (13.0%)	9 (1.6%)	**< 0.001**		3 (1.4%)	5 (1.5%)	0.893	
**Interested in receiving further information…** (mean value ± SD on a scale from 1 to 5)								
…about the Coronavirus	1.0 ± 1.2	3.8 ± 1.7	**< 0.001**	20.9** (2.8-155.5)	2.4 ± 1.7	3.5 ± 1.5	**< 0.001**	2.9** (2.0-4.2)

* The number of analysed answers differs per question category deviating from the basic population. ** OR remained significant after adjustment for age, sex, ethnicity and education Significant findings are in bold.

For all seafarers the shipping company 370 (68.1%), the ship’s personnel 314 (59.1%) and the international organizations 156 (29.8%) were the most frequently used sources of information on a regular basis. No seafarer added another relevant information channel in free text. Comparing officers with ratings, a significant (p = < 0.001) difference was observed as the regular information transfer of ratings more often relies on sources ashore – such as port workers (23.8%), port medical services (20.1%) and Seamen’s missions (13.9%). However, ratings used also significantly more often ships-related sources (67.2% ships personnel) and internet-based information channels (34.5% international organisations). All results are shown in Table 6.

**Table 6:** Regularly used channels for COVID-19 information among ratings and officers.

Information on COVID-19 provided regularly by…	Seafarers (*n* = 583)	Officers (*n* = 215)	Ratings (*n* = 337)	*p*
Shipping company	370 (68.1%)	141 (65.6%)	229 (69.8%)	0.099
Ship’s personnel	314 (59.1%)	95 (46.3%)	219 (67.2%)	**< 0.001**
Port workers	106 (20.1%)	29 (14.3%)	77 (23.8%)	**< 0.001**
Port medical services	91 (17.3%)	26 (12.8%)	65 (20.1%)	**< 0.001**
Seamen’s Mission	59 (11.2%)	14 (6.9%)	45 (13.9%)	**< 0.001**
International organisations (e.g. IMO, ITF, SIU)	156 (29.8%)	46 (22.5%)	110 (34.5%)	**0.012**

* The number of analysed answers differs per question category deviating from the basic population. Significant findings are in bold.

## Discussion

At the beginning of 2022, after more than two years of the pandemic, the rate of new COVID-19 infections peaked worldwide^35^. The particularly difficult situation for seafarers (separated from their homes for several months at one time) and their families on land meant an enormous psychosocial burden. It has been widely described that the pandemic had a negative impact on the mental health of seafarers and that certain strategies can improve it^15–17^. In this situation, vaccinations and information about virus transmission as well as preventive protective options were particularly important. As there is little information on vaccination rates on board, the psychosocial burden on seafarers and the communication channels and information media used in shipping, this was the subject of the present study – with a focus on the period of February 2022.

In order to ensure the transportation of vital goods by the shipping industry in times of a pandemic, the health and thus also the vaccination of seafarers is a crucial point^36^. To date, there has been no international tracking of seafarer vaccination, only a survey by the Global Maritime Forum^8,9^. The present study found that 80.7% of the surveyed ship crews were vaccinated by February 2022. The difference to the 89% recorded by the Global Maritime Forum in July 2022 is even more alarming. There is also a difference in the trend compared to the office workers with a vaccination rate of 95.8%. In addition, the vaccine doses received by seafarers are different compared to the office workers: Office workers were 31 times (CI 12.0-81.9) more likely to receive three doses than seafarers, then reported as primary immunization with a booster^34^. Although there were vaccines that originally only required one dose^37^, this vaccine was used in both groups (Table 7) and by February 2022 there were already recommendations to improve this particular vaccination status with further doses. This confirms the assumption of a low vaccination rate among seafarers – even in early 2022^8,9^. When examining the reasons for non-vaccination of seafarers, the most common qualitive responses were the lack of availability of vaccination in their home country (41 seafarers) or the non-occurrence of a vaccination by then, once again highlighting the importance of facilitating seafarers’ access to vaccination during a pandemic.

Accordingly, shipping crews craved any vaccination opportunity they could get. This was often organized from the bottom up by seafarer’s unions and shore-based welfare organizations^9^, resulting in a lack of uniform vaccination standards within the collective on board. As a result, seafarers received a wide variety of vaccines compared to shore-based workers: While the office workers in the present study received only four different vaccines, the seafarers were vaccinated with eight different vaccines, as shown in Table 7. This finding further accentuates the lack of a sufficient vaccination program for seafarers as key workers during the COVID-19 pandemic^9^.

**Table 7:** Covid-19 vaccines used in office workers and seafarers.

COVID-19 vaccines	Office workers (*n* doses = 62)	Seafarers (*n* doses = 830)
Comirnaty (BioNTech/Pfizer)	37 (60%)	277 (33.4%)
Spikevax (Moderna)	17 (27.4%)	131 (15.6%)
Janssen (Johnson & Johnson)	4 (6.5%)	128 (15.4%)
Sinopharm	0	119 (14.3%)
CoronaVac	0	87 (10.5%)
Vaxzervria (AstraZeneca)	4 (6.5%)	55 (6.6%)
Sputnik	0	19 (2.3%)
Covishield	0	14 (1.7%)

The heterogeneity of vaccines used illustrates the great diversity and complexity of the strategy for coping with the pandemic demands in seafaring. This can certainly lead to uncertainty and psychosocial problems among the seafaring personnel, in addition to the lower vaccinations rate and generally disadvantaged working condition compared to non-seafarers. Therefore, it is of great importance to assess the extent of psychosocial stress on ship crews during the pandemic and to improve the information channels for dealing with virus-related health hazards.

This population showed a significant fear of health consequences in the FCQ^28,29^ in contrast to the office workers (mean value 23.4 ± 5.7 in office workers vs. 28.7 ± 6.6 in seafarers, *p* < 0.001, crude OR 3.8, CI 1.4–10.4). This result can also be transferred to the social and financial burden of the pandemic on seafarers (*p* = 0.005, *p* < 0.001). In particular, shipping crews are also significantly more often worried about the outbreak, about family and friends and about their personal health than office workers. They are more likely to feel that health authorities are not doing enough (Table 3). This is easily explained by their working conditions at sea compared to life on land with home office, curfews and early vaccination. Although the pandemic is already two years old, these findings are consistent with those of Coutroubis et al., who surveyed 400 seafarers at sea in March 2020. Of these, 84% said they were worried about future employment, 95% that someone at sea could fall ill, 96% that someone could bring the virus on board, 80% that they would be more isolated than before and 86% about travelling home after their contract^20^.

This study can also show the results differentiating by rank: Ratings were 2.5 times more often afraid of coronavirus and its health consequences than officers (26.2 ± 6.5 in officers vs. 30.4 ± 6.1 in ratings, *p* < 0.001, crude OR 3.7, CI 2.6–5.4). In addition, they were two to three times more likely than officers to agree with every question of the FCQ and perceived a higher social and financial burden from the pandemic by the same rate. These results may be due to the closer working conditions of ratings, the poorer accessibility of information and the longer contract duration of around 9 months compared to officers at around 3 months at a time. Furthermore, officers have a higher level of education, which may be associated with a better ability to understand the complexity of a pandemic and a high degree of individual freedom due to a greater scope for decision-making and action. This additionally indicated a probable higher resilience, which was also objectified significantly more often in officers than in ratings by Janssen et al. (OR 1.74; 95% CI 1.27–2.39)^38^. These aspects underline the impact of a pandemic on the seafarers due to their working conditions and emphasize the importance of support to address their concerns^16^.

In order to deal with fear and minimize the risk of infection in the context of a pandemic, it is essential to have knowledge on this topic^25,31^. Therefore, this study aimed to assess the subjectively perceived sufficiency of knowledge and ways of knowledge transfer among shipping crews with regard to COVID-19. With 82.7% of the seafarers feeling (very) well informed on COVID-19 issues and 75.7% on COVID-19 vaccination, these results are in line with Battineni et al. who found that 97% of seafarers had sufficient knowledge on COVID-19 in a questionnaire in 2020^26^. Nevertheless, after two years of the pandemic there were apparently few seafarers without sufficient knowledge of COVID-19. Furthermore, although seafarers were significantly less likely to report not having received compared to office workers (13% office workers, 1.6% seafarers, *p* < 0.001), they were 20.9 times more likely to be interested in further information (CI 2.8-155.5). This could also be explained by the more exposed and more precarious situation of seafarers. This phenomenon was also evident in the results of officers and ratings: While ratings showed almost twice as high satisfaction with their knowledge about COVID-19 and vaccination compared to officers (*p* < 0.001; *p* < 0.001), they were 2.9 times more interested in receiving further information (CI 2.0-4.2). Various reasons for higher perceived anxiety mentioned may apply here. All in all, this result indicates that the dissemination of information on pandemic issues to ship crews must be intensified in future crisis.

To be able to make recommendations for the way in which information is passed on, this study also analysed the communication channels and information media used by seafarers. While the majority of information was obtained from the shipping company (68.1%), the ship’s personnel (59.1%) and international organisations (29.8%), there was a difference between the information channels used by officers and ratings: Ratings were significantly more likely to be informed through contacts in port – 28.3% through port workers, 20.1% through port medical services and 13.9% through Seamen Missions. In terms of information, ratings used messenger platforms significantly more often and mails less often than officers (*p* < 0.001; *p* < 0.001). Differences in the use of communication channels and information media between the occupational groups can be explained, among others, by the fact that officers often have internet access in their cabin, their higher salary enables paid internet access on the high seas and a higher level of education possibly offers better information transfer. These findings point to the importance of knowledge transfer by the shipping company and internet-based international organisation, as well as the relevance of shore-based contacts for the ratings. Furthermore, these differences between occupational groups need to be taken into account when planning and optimizing information channels and resources in the context of general pandemic education.

Insufficient information not only creates fear among seafarers, but also contributes to misconceptions and irrational beliefs. Fear of side effects and scepticism about the effectiveness of the vaccination were cited twelve times in this study as reasons for not getting vaccinated. It can only be speculated at this point whether sufficient scientifically based information would have subsequently changed the attitude of these few participants towards their willingness to be vaccinated.

### Strengths and limitations

This study was one of the few to examine the vaccination rate of seafarers, the psychosocial stress situation and preventive education methods during the world’s largest peak of new COVID-19 infections. The study group consisted of a significant number of seafarers who worked on a ship during the COVID-19 pandemic (583 seafarers on 33 ships). A significant recall bias can therefore be ruled out in principle. With a participation rate of 94.6% and no exclusion criteria, it can be assumed that the study results are highly representative of shipping crews. Even if it is not possible to generalize the statements of this study, it can be assumed that the working conditions of seafarers are very similar due to the international regulations. Furthermore, the great willingness to participate in this survey indicates a great interest in researching and improving their situation.

In addition to the suitable study group of seafarers and the more detailed results on COVID-19 anxiety, knowledge and vaccination in contrast to previous publications, this study is limited by the rather small group of participating office workers. It can lead to unbalanced comparisons that impair the statistical significance. Nevertheless, this group served as an internal reference sample and enabled a comparison of COVID-19 vaccination rates, fears, knowledge and information. The data represents a specific phase during the pandemic and can hardly map all phases. However, the investigation was carried out specifically at a time of a very high global infection rate and these phases will occur in every pandemic. Although sufficient language skills are assumed for seafarers, a scientific questionnaire in English could have been a language barrier for multinational crews. Therefore, no scientific questionnaire was used other than the FCQ and the simplest possible language level was applied. As there were no exclusion criteria, the lack of data for individual questions has an impact on consistency. Another limitation is, that this study only asked about the information channels and media, but not about their validity and reliability. Therefore, the accuracy of the information received by the crew cannot be assessed. This question should be the subject of further research with regard to health literacy in the maritime sector.

## Conclusion

These results provide new information about the high mental, social and financial burden on seafarers during the COVID-19 pandemic. In addition, the study shows that ship crews were highly interested in further information about the coronavirus compared to office workers, suggesting that information on pandemic issues needs to be further intensified during a pandemic. While there is limited data on seafarer vaccination rates, this research provides data on vaccination status during the greatest peak of new COVID-19 infections in February 2022. Overall, these findings highlight the importance of supporting seafarers in future crises and the need to improve international strategies for treating seafarers as key workers and better coordinated vaccinations processes.

## Supplementary Information

Below is the link to the electronic supplementary material.

Supplementary Material 1

## Data Availability

The datasets used and/or analysed during the current study are available from the corresponding author on reasonable request.

## References

[1] World Health Organization. Coronavirus disease (COVID-19) pandemic. (2025). https://www.who.int/europe/emergencies/situations/covid-19

[2] Worldometer Coronavirus statistics. (2024). https://www.worldometers.info/coronavirus

[3] International Chamber of Shipping. Shipping and world trade: Global supply and demand for seafarers. (2024). https://www.ics-shipping.org/shipping-fact/shipping-and-world-trade-global-supply-and-demand-for-seafarers

[4] European Maritime Safety Agency. Discover the EU maritime profile. https://www.emsa.europa.eu/eumaritimeprofile.html#:~:text=Approximately%2090%25%20 (2024). of%20world%20trade,as%20trade%2 C%20tourism%2 C%20fisheries.

[5] United Nations. General Assembly. International cooperation to address challenges faced by seafarers as a result of the COVID-19 pandemic to support global supply chains 2020. (2020). https://documents.un.org/doc/undoc/ltd/n20/332/14/pdf/n2033214.pdf

[6] International Maritime Organization. Coronavirus (COVID-19) - Joint statement calling on all governments to prioritzie COVID-19 vaccination for seafarers and aircrew. (2021). https://wwwcdn.imo.org/localresources/en/MediaCentre/Documents/Circular%20Letter%20No.4204-Add.38%20-%20Coronavirus%20(Covid-19)%20-%20Joint%20Statement%20Calling%20On%20All%20GovernmentsTo%20Prioritize%20Covid-19… Secretariat).pdf.

[7] Internation Chamber of Shipping. Coronavirus (COVID-19). Roadmap for vaccination of international seafarers. (2021). https://www.ics-shipping.org/wp-content/uploads/2021/05/Coronavirus-COVID-19-Roadmap-for-Vaccination-of-International-Seafarers-1.pdf

[8] Han, W., Chen, J., Wei, K., Shi, J. & Jia, G. International crew changes amid global pandemic outbreaks: key issues and system innovations. *Mar. Policy*. **147**, 105342 (2023). 36312743 10.1016/j.marpol.2022.105342 PMC9595386

[9] Lucas, D. et al. Vaccinating international seafarers during the COVID-19 pandemic. *Lancet Glob Health*. **12** (1), e166–e9 (2024). 38097286 10.1016/S2214-109X(23)00486-2

[10] Battineni, G., Kumar, S., Mittal, M. & Amenta, F. COVID-19 vaccine on board ships: current and future implications of seafarers. *Int. Marit Health*. **72** (1), 76–77 (2021). 33829476 10.5603/IMH.2021.0010

[11] International Chamber of Shipping. Annual review 2020. Heroes at sea. (2020). https://www.ics-shipping.org/wp-content/uploads/2020/11/annual-review-2020-final-compressed.pdf

[12] Oldenburg, M., Baur, X. & Schlaich, C. Occupational risks and challenges of seafaring. *J. Occup. Health*. **52** (5), 249–256 (2010). 20661002 10.1539/joh.k10004

[13] Nittari, G., Gibelli, F., Bailo, P., Sirignano, A. & Ricci, G. Factors affecting mental health of seafarers on board merchant ships: a systematic review. *Rev. Environ. Health*. **39** (1), 151–160 (2024). 36302371 10.1515/reveh-2021-0070

[14] Shan, D. Occupational safety and health challenges for maritime key workers in the global COVID-19 pandemic. *Int. Labour Rev.* **161** (2), 267–287 (2022). 34548682 10.1111/ilr.12220 PMC8444828

[15] De Beukelaer, C. COVID-19 border closures cause humanitarian crew change crisis at sea. *Mar. Policy*. **132**, 104661 (2021). 34602714 10.1016/j.marpol.2021.104661 PMC8462811

[16] Hayes-Mejia, R. & Stafstrom, M. Psychosocial work environment and mental health among the global workforce of seafarers in the wake of the COVID-19 pandemic. *BMC Public. Health*. **23** (1), 2151 (2023). 37924109 10.1186/s12889-023-17035-2 PMC10623868

[17] Brooks, S. K. & Greenberg, N. Mental health and wellbeing of seafaring personnel during COVID-19: scoping review. *J. Occup. Health*. **64** (1), e12361 (2022). 36134469 10.1002/1348-9585.12361 PMC9494025

[18] Pauksztat, B., Grech, M. R. & Kitada, M. The impact of the COVID-19 pandemic on seafarers’ mental health and chronic fatigue: beneficial effects of onboard peer support, external support and internet access. *Mar. Policy*. **137**, 104942 (2022). 35013636 10.1016/j.marpol.2021.104942 PMC8732879

[19] Stannard, S. COVID-19 in the maritime setting: the challenges, regulations and the international response. *Int. Marit Health*. **71** (2), 85–90 (2020). 32604450 10.5603/IMH.2020.0016

[20] Coutroubis, A. D., Menelaou, A. A. & Adami, E. H. Impact of coronavirus disease (COVID-19) on seafarer’s life and well-being. *Int. J. Trop. Dis. Health*. **41** (21), 16–27 (2020).

[21] Ozabor, F., Efe, S. I. & Kpang, M. B. T. Obisesan, A. social and economic wellbeing of seafarers across coastal Nigeria amidst Corona virus disease. *Heliyon* **9**(8), e18275 (2023). 10.1016/j.heliyon.2023.e18275 PMC10407044 37560696

[22] International Transport Workers’ Federation. Beyond the limit of safe shipping. (2020). https://www.itfglobal.org/en/news/beyond-limit-safe-shipping-itf-general-secretarys-un-address

[23] Dengler, D. et al. [Prevention and management of COVID-19 outbreaks on merchant ships]. *Zentralbl Arbeitsmed Arbeitsschutz Ergon.* **71** (6), 296–304 (2021). 34456517 10.1007/s40664-021-00440-y PMC8385476

[24] Kordsmeyer, A. C. et al. Systematic review on outbreaks of SARS-CoV-2 on cruise, navy and cargo ships. *Int J. Environ. Res. Public. Health* **18**(10), 5195 (2021). 10.3390/ijerph18105195 PMC8153346 34068311

[25] Psychosocial Centre of the International Federation of Red Cross and Red Crescent Societies. Mental health and psychosocial support for staff, volunteers and communities in an outbreak of novel coronavirus. (2020). https://pscentre.org/wp-content/uploads/2020/02/MHPSS-in-nCoV-2020_ENG-1.pdf

[26] Battineni, G., Sagaro, G. G., Chintalapudi, N., Di Canio, M. & Amenta, F. Assessment of awareness and knowledge on novel coronavirus (COVID-19) pandemic among seafarers. *Healthcare (Basel)* **9**(2), 120 (2021). 10.3390/healthcare9020120 PMC7912131 33503921

[27] Williams, B. A., Jones, C. H., Welch, V. & True, J. M. Outlook of pandemic preparedness in a post-COVID-19 world. *NPJ Vaccines*. **8** (1), 178 (2023). 37985781 10.1038/s41541-023-00773-0 PMC10662147

[28] Mertens, G., Duijndam, S., Smeets, T. & Lodder, P. The latent and item structure of COVID-19 fear: A comparison of four COVID-19 fear questionnaires using SEM and network analyses. *J. Anxiety Disord*. **81**, 102415 (2021). 33962142 10.1016/j.janxdis.2021.102415 PMC8091728

[29] Mertens, G., Gerritsen, L., Duijndam, S., Salemink, E. & Engelhard, I. M. Fear of the coronavirus (COVID-19): predictors in an online study conducted in March 2020. *J. Anxiety Disord*. **74**, 102258 (2020). 32569905 10.1016/j.janxdis.2020.102258 PMC7286280

[30] Vos, L. M. W., Habibovic, M., Nyklicek, I., Smeets, T. & Mertens, G. Optimism, mindfulness, and resilience as potential protective factors for the mental health consequences of fear of the coronavirus. *Psychiatry Res.* **300**, 113927 (2021). 33848964 10.1016/j.psychres.2021.113927 PMC9755114

[31] Skoda, E. M. et al. Psychological burden of healthcare professionals in Germany during the acute phase of the COVID-19 pandemic: differences and similarities in the international context. *J. Public. Health (Oxf)*. **42** (4), 688–695 (2020). 32766787 10.1093/pubmed/fdaa124 PMC7454781

[32] Rasch, D., Kubinger, K. D. & Moder, K. The two-sample t test: pre-testing its assumptions does not pay off. *Stat. Papers*. **52**, 219–231 (2011).

[33] Ruxton, G. D. The unequal variance t-test is an underused alternative to student’s t-test and the Mann–Whitney U test. *Behav. Ecol.* **17** (4), 688–690 (2006).

[34] Robert Koch Institut. Epidemiologisches Bulletin 03/2022. (2022). https://www.rki.de/DE/Content/Infekt/EpidBull/Archiv/2022/03/Art_02.html

[35] World Health Organization. WHO COVID-19 dashboard. (2025). https://data.who.int/dashboards/covid19/cases?n=c

[36] Schlaich, C. C., Lucas, K., Sydow, S., Beyer, E. & Faesecke, K. P. Procedural aspects of COVID-19 vaccinations for seafarers on ocean-going vessels. *Int. Marit Health*. **72** (3), 179–182 (2021). 34604986 10.5603/MH.2021.0034

[37] Stiftung Gesundheitswissen. Johnson & Johnson-Impfstoff: Fragen und Antworten zu Jcovden (früher: Janssen). (2022). https://www.stiftung-gesundheitswissen.de/wissen/covid-19-impfung/faq-zum-covid-19-impfstoff-janssen-johnson-johnson

[38] Janssen, W., Jensen, H. J., Harth, V. & Oldenburg, M. Systematic review: measurement methods and concept of resilience among seafarers. *J. Health Care*. **61**, 1–12 (2024). 10.1177/00469580231221288 PMC10798076 38240089

[39] Sharma, A. et al. A Consensus-Based checklist for reporting of survey studies (CROSS). *J. Gen. Intern. Med.* **36** (10), 3179–3187 (2021). 33886027 10.1007/s11606-021-06737-1 PMC8481359

