# Supplementary material for: Psychosocial burden, vaccination status and preventive information options for seafarers during the COVID-19 pandemic
Source: Sci Rep. 2025 Sep 23;15:32682. doi: 10.1038/s41598-025-20616-3 (PMC12457601; doi:10.1038/s41598-025-20616-3)
Supplement: Supplementary file 1 — Supplementary Material 1 [file 41598_2025_20616_MOESM1_ESM.pdf]

Psychosocial burden, vaccination status and preventive information options for seafarers during the COVID-19 pandemic

Nora M. Puls*, Benedict Oldenburg, Chiara Reck, Dorothee Dengler, Lukas Belz, Lorenz Scheit, Volker Harth, Marcus Oldenburg

Supplementary Table S1. Checklist for Reporting Of Survey Studies (CROSS).

Section/topic	Item	Item description	Reported on page #
**Title and abstract**			
Title and abstract	1a	State the word "survey" along with a commonly used term in title or abstract to introduce the study's design.	1
	1b	Provide an informative summary in the abstract, covering background, objectives, methods, findings/results, interpretation/discussion, and conclusions.	1-2
**Introduction**			
Background	2	Provide a background about the rationale of study, what has been previously done, and why this survey is needed.	2-3
Purpose/aim	3	Identify specific purposes, aims, goals, or objectives of the study.	3
**Methods**			
Study design	4	Specify the study design in the methods section with a commonly used term (e.g., cross-sectional or longitudinal).	3
	5a	Describe the questionnaire (e.g., number of sections, number of questions, number and names of instruments used).	3-5
	5b	Describe all questionnaire instruments that were used in the survey to measure particular concepts. Report target population, reported validity and reliability information, scoring/classification procedure, and reference links (if any).	4-5
Data collection methods	5c	Provide information on pretesting of the questionnaire, if performed (in the article or in an online supplement). Report the method of pretesting, number of times questionnaire was pre-tested, number and demographics of participants used for pretesting, and the level of similarity of demographics between pre-testing participants and sample population.	N/A
	5d	Questionnaire if possible, should be fully provided (in the article, or as appendices or as an online supplement).	S2
Sample characteristics	6a	Describe the study population (i.e., background, locations, eligibility criteria for participant inclusion in survey, exclusion criteria).	4

	6b	Describe the sampling techniques used (e.g., single stage or multistage sampling, simple random sampling, stratified sampling, cluster sampling, convenience sampling). Specify the locations of sample participants whenever clustered sampling was applied.	N/A
	6c	Provide information on sample size, along with details of sample size calculation.	N/A
	6d	Describe how representative the sample is of the study population (or target population if possible), particularly for population-based surveys.	N/A
Survey administration	7a	Provide information on modes of questionnaire administration, including the type and number of contacts, the location where the survey was conducted (e.g., outpatient room or by use of online tools, such as SurveyMonkey).	4
	7b	Provide information of survey's time frame, such as periods of recruitment, exposure, and follow-up days.	3-4
	7c	Provide information on the entry process: For non-web-based surveys, provide approaches to minimize human error in data entry. For web-based surveys, provide approaches to prevent "multiple participation" of participants.	4
Study preparation	8	Describe any preparation process before conducting the survey (e.g., interviewers' training process, advertising the survey).	N/A
Ethical considerations	9a	Provide information on ethical approval for the survey if obtained, including informed consent, institutional review board [IRB] approval, Helsinki declaration, and good clinical practice [GCP] declaration (as appropriate).	4
	9c	Provide information about survey anonymity and confidentiality and describe what mechanisms were used to protect unauthorized access.	4
Statistical analysis	10a	Describe statistical methods and analytical approach. Report the statistical software that was used for data analysis.	5-6
	10b	Report any modification of variables used in the analysis, along with reference (if available).	N/A
	10c	Report details about how missing data was handled. Include rate of missing items, missing data mechanism (i.e., missing completely at random [MCAR], missing at random [MAR] or missing not at random [MNAR]) and methods used to deal with missing data (e.g., multiple imputation).	4
	10d	State how non-response error was addressed.	4
	10e	For longitudinal surveys, state how loss to follow-up was addressed.	N/A
	10f	Indicate whether any methods such as weighting of items or propensity scores have been used to adjust for non-representativeness of the sample.	N/A

	10g	Describe any sensitivity analysis conducted.	N/A
**Results**			
Respondent characteristics	11a	Report numbers of individuals at each stage of the study. Consider using a flow diagram, if possible.	6
	11b	Provide reasons for non-participation at each stage, if possible.	6
	11c	Report response rate, present the definition of response rate or the formula used to calculate response rate.	6
	11d	Provide information to define how unique visitors are determined. Report number of unique visitors along with relevant proportions (e.g., view proportion, participation proportion, completion proportion).	N/A
Descriptive results	12	Provide characteristics of study participants, as well as information on potential confounders and assessed outcomes.	6-7
Main findings	13a	Give unadjusted estimates and, if applicable, confounder-adjusted estimates along with 95% confidence intervals and p-values.	6-14
	13b	For multivariable analysis, provide information on the model building process, model fit statistics, and model assumptions (as appropriate).	N/A
	13c	Provide details about any sensitivity analysis performed. If there are considerable amount of missing data, report sensitivity analyses comparing the results of complete cases with that of the imputed dataset (if possible).	N/A
**Discussion**			
Limitations	14	Discuss the limitations of the study, considering sources of potential biases and imprecisions, such as non-representativeness of sample, study design, important uncontrolled confounders.	18
Interpretations	15	Give a cautious overall interpretation of results, based on potential biases and imprecisions and suggest areas for future research.	19
Generalizability	16	Discuss the external validity of the results.	14-17
**Other sections**			
Role of the funding source	17	State whether any funding organization has had any roles in the survey's design, implementation, and analysis.	23
Conflict of interest	18	Declare any potential conflict of interest.	23
Acknowledgements	19	Provide names of organizations/persons that are acknowledged along with their contribution to the research.	Provided at submission

Supplementary Material S2. Questionnaire Health Promotion for Seafarers

Health Promotion for Seafarers

This questionnaire serves to evaluate your opinion concerning your needs and interests on how to improve the living and working situation on board. Based on your answers we plan to develop and test some measures on board that could raise your health and wellbeing during your shipboard stay.

We ask you to please participate in this voluntary survey. Your answers will be collected and processed **confidentially and anonymously by the scientists of the Institute for Occupational Medicine and Maritime Medicine (ZfAM) that is affiliated to the University Medical Centre Hamburg-Eppendorf (UKE)**. Therefore, your name will not be recorded. Please answer each question, even if it doesn´t seem to fit 100% to the maritime setting by looking at your average shipboard experience. The more information you give us the better and more specific health promotion can be planned. Thank you!

COVID-19

14. **Fear of the Coronavirus**
Please select the extent to which the following thoughts, feelings and behaviors apply to you.

		**Strongly disagree**	**Disagree**	**Neutral**	**Agree**	**Strongly agree**
14.1	I am very worried about the corona virus outbreak					
14.2	I am taking precautions to prevent infection (e.g., washing hands, avoiding contact with people, avoiding door handles)					
14.3	I am constantly following all news updates regarding the virus					
14.4	I have stocked up on supplies to prepare for problems related to the coronavirus outbreak					
14.5	For my personal health I find the virus to be much more dangerous than the seasonal					
14.6	I feel that the health authorities are not doing enough to deal with the virus					
14.7	I am worried that friends or family will be infected					
14.8	I take more precautions compared to most people to not become infected					

15. **How much do you feel burdened by the social consequences of the corona pandemic?**

☐	☐	☐	☐	☐	☐
0<br>Not at all	1	2	3	4	5<br>Extremely

16. **How much do you feel burdened by the financial consequences of the corona pandemic?**

☐	☐	☐	☐	☐	☐
0<br>Not at all	1	2	3	4	5<br>Extremely

17. **Do you feel sufficiently informed related to COVID-19 issues?**
☐ Very well informed ☐ Well informed ☐ Fairly informed
☐ Poorly informed ☐ Very poorly informed

18. **Do you feel sufficiently informed related to COVID-19 vaccines?**
☐ Very well informed ☐ Well informed ☐ Fairly informed
☐ Poorly informed ☐ Very poorly informed

19. **Who from your work environment provides you with information on COVID-19 during your service on board?**

A) Shipping company:
☐ Regularly ☐ Sometimes ☐ Never

B) Ship's personnel:
☐ Regularly ☐ Sometimes ☐ Never

C) Port workers:
☐ Regularly ☐ Sometimes ☐ Never

D) Port medical services:
☐ Regularly ☐ Sometimes ☐ Never

E) Seamen's Mission:
☐ Regularly ☐ Sometimes ☐ Never

F) International organizations (e.g. IMO, ITF, SIU):
☐ Regularly ☐ Sometimes ☐ Never

G) Other, please specify:__________________

20. **What communication channels are used to disseminate information on COVID-19 by your work environment?** *(more than one answer possible)*
☐ Person-to-person communications
☐ E-mails
☐ Flyers/leaflets
☐ Web-based trainings
☐ Messenger platforms (e.g. WhatsApp, Telegram, etc.)
☐ Other, please specify ______________________
☐ I didn’t receive information

21. **Are you interested in receiving further information about the Coronavirus?**

☐	☐	☐	☐	☐	☐
0	1	2	3	4	5
No, not at all interested					Yes, extremely interested

Demographic and occupational data

26. **What is your rank?**

☐ Nautical officer ☐ Technical officer/Electrician ☐ Rating engine ☐ Rating deck
☐ Galley staff ☐ Cadet ☐ Other______________

27. **What is your highest educational degree (please choose national equivalent)?**
☐No degree ☐Basic/Middle school degree ☐High school degree ☐University degree

28. **What is your age?**

☐19 or younger ☐20-29 years ☐30-39 years ☐40-49 years ☐50-59 years ☐60 or older

31. **How long have you worked as a seafarer? years** ☐☐ years

32. **What continent are you from?**

☐ Europe ☐ Asia ☐ Africa ☐ North America ☐ South America ☐ Australia

33. **Are you...?** sex ☐ Male ☐ Female ☐ Other

37. **Are you vaccinated against the Coronavirus (COVID-19)?** ☐ Yes ☐ No

37.1 **If yes, how many vaccine doses have you been given?** _________ doses

37.2 **If yes, which vaccine did you get?** If you got different vaccines, please name all of them (e.g.: Johnson&Johnson, Astra Zeneca, Sputnik V, Moderna, Biontech, ...).

First dose: ________________________ Second dose: ________________________

Third dose: ________________________ Further doses: ________________________

37.3 **If no, what was the reason not to be vaccinated against the Coronavirus?**

____

Thank you very much for your participation!
